# Avaliação da efetividade da intervenção para aprimoramento do Sistema de Informações sobre Mortalidade em Pernambuco, Brasil: um estudo quase-experimental

**DOI:** 10.1590/0102-311XPT077523

**Published:** 2024-01-08

**Authors:** Barbara de Queiroz Figueirôa, Pedro Israel Cabral de Lira, Lygia Carmen de Moraes Vanderlei, Suely Arruda Vidal, Paulo Germano de Frias

**Affiliations:** 1 Programa de Pós-graduação em Saúde da Criança e do Adolescente, Universidade Federal de Pernambuco, Recife, Brasil.; 2 Secretaria Estadual de Saúde de Pernambuco, Recife, Brasil.; 3 Programa de Pós-graduação em Avaliação em Saúde, Instituto de Medicina Integral Prof. Fernando Figueira, Recife, Brasil.

**Keywords:** Registros de Mortalidade, Sistemas de Informação em Saúde, Avaliação em Saúde, Estatísticas Vitais, Mortality Registries, Health Information Systems, Health Evaluation, Vital Statistics, Registros de Mortalidad, Sistemas de Información en Salud, Evaluación en Salud, Estadísticas Vitales

## Abstract

Avaliou-se a influência da variação da implantação do Sistema de Informações sobre Mortalidade (SIM) nos resultados antes e depois da intervenção para aprimoramento do sistema em Pernambuco, Brasil. Foram explicitados o modelo lógico e a matriz de indicadores e julgamento do SIM. Coletaram-se dados primários nos 184 municípios e dados secundários na base do sistema. Obteve-se o grau de implantação (GI) a partir de indicadores de estrutura e processo, posteriormente relacionado aos de resultado, com base no modelo. A intervenção foi direcionada às falhas identificadas e se desenvolveu mediante etapas estratégicas. Foi calculado o percentual de variação anual do GI e dos resultados antes e depois da intervenção. Classificou-se o SIM como parcialmente implantado nas avaliações pré (70,6%) e pós-intervenção (73,1%), com incrementos em todos os componentes. As Regiões de Saúde seguiram a mesma classificação do âmbito estadual, excetuando-se a XII (80,3%), com escore implantado, após a intervenção. Cobertura do sistema, óbitos com causa básica definida, transferência mensal e envio oportuno de dados situaram-se acima de 90% nas duas avaliações. Houve melhora na completude das Declarações de Óbito infantil e no registro oportuno de eventos notificáveis. O fortalecimento da gestão e operacionalização do SIM por meio de intervenções aplicadas no contexto de produção dos dados pode aprimorar os resultados do sistema.

## Abstract (en)

This study evaluated the influence of the variation in the implementation of the Brazilian Mortality Information System (SIM) on the results, before and after the intervention to improve the system in Pernambuco, Brazil. The SIM logical model and matrix of indicators and assessment were described, primary data were collected from the 184 municipalities and secondary data were collected from the system database. The degree of implementation (DI) was obtained from the indicators of structure and process, and then related to result indicators, based on the model. The intervention was directed at the shortcomings identified, and developed using strategic stages. The percentage of annual variation of the DI and the results before and after the intervention were calculated. The SIM was classified as partially implemented in the pre- (70.6%) and post-intervention (73.1%) evaluations, with increments in all components. The Health Regions followed the same classification of the state level, except for XII (80.3%), regarding implemented score after the intervention. The coverage of the system; deaths with a defined underlying cause; monthly transfer; and timely submission of data were above 90% in both evaluations. There was an improvement in the completeness of infant Death Certificates and in the timely recording of notifiable events. Strengthening the management and operationalization of the SIM with interventions applied to data registration can improve the system’s results.

## Resumen

Se evaluó la influencia de la variación de la implantación del Sistema de Informaciones sobre la Mortalidad (SIM) en los resultados, antes y después de la intervención para mejorar el sistema en Pernambuco, Brasil. Se explicitaron el modelo lógico y la matriz de indicadores y juicio del SIM, recolectando datos primarios en los 184 municipios y datos secundarios en la base del sistema. Se obtuvo el grado de implantación (GI) a partir de indicadores de estructura y proceso, posteriormente, relacionado con los indicadores de resultado, basado en el modelo. La intervención se dirigió a los fallos identificados y se desarrolló a través de etapas estratégicas. Se calcularon el porcentaje de variación anual del GI y de los resultados antes y después de la intervención. El SIM se clasificó como parcialmente implantado en las evaluaciones previas (70,6%) y posteriores a la intervención (73,1%) con aumento en todos los componentes. Las Regiones de Salud han seguido la misma clasificación del ámbito estatal, salvo la XII (80,3%), con puntaje implantado, después de la intervención. La cobertura del sistema; óbitos debido a causa básica definida; transferencia mensual y envío oportuno de datos, estuvieron por encima del 90% en ambas evaluaciones. Hubo una mejora en la exhaustividad de los Certificados de Muerte infantiles y en el registro oportuno de eventos notificables. Fortalecer la gestión y la implementación del SIM a través de intervenciones aplicadas en el contexto de producción de datos puede mejorar los resultados del sistema.

## Introdução

Os sistemas de informações vitais de registro contínuo são imprescindíveis para a constituição de diversos indicadores sociossanitários. Sua utilidade no monitoramento de importantes pactos nacionais e internacionais acentuou a necessidade da obtenção de dados fidedignos e incentivou as discussões sobre sua acurácia ^1^. No entanto, limitações para a elaboração de indicadores diante da reduzida adequação desses sistemas impulsionaram a realização de avaliações para auxiliar a qualificação dos seus dados ^2^^,^^3^.

No Brasil, essas experiências ocorreram de maneira isolada e assistemática, fundamentando-se, essencialmente, na análise dos atributos de qualidade ^3^. Na última década, estudos avaliativos consideraram as condições estruturais e processuais no contexto de produção da informação e ampliaram a perspectiva analítica dos sistemas de registros vitais do Ministério da Saúde, apontando fatores explicativos sobre os resultados esperados, com base nos seus elementos regulamentadores ^4^^,^^5^^,^^6^^,^^7^. Entretanto, ainda que essas experiências tenham ampliado o conhecimento sobre falhas na produção dos dados dos sistemas de informações vitais ^4^^,^^5^^,^^6^^,^^7^, não há relatos a respeito da sua aplicabilidade à rotina do Sistema de Informações sobre Mortalidade (SIM).

Ao longo de décadas, diversas ações foram empreendidas para aprimorar os sistemas de registros contínuos de óbitos, em particular o SIM, repercutindo na qualidade dos dados, embora persistam obstáculos à sua plena utilização em algumas regiões do país ^8^^,^^9^^,^^10^. As falhas na acurácia dos sistemas são continuamente identificadas sobretudo no Norte e Nordeste brasileiro ^11^. Por outro lado, o desconhecimento de fatores explicativos e de quais intervenções têm maior potencial para reduzir esses problemas reflete carências avaliativas que dificultam o direcionamento de medidas para ampliar a qualidade do sistema no seu contexto de operacionalização.

Analisar intervenções na perspectiva do caráter complexo e multidimensional do ambiente necessita de alternativas metodológicas adequadas para sua fundamentação ^12^. Assim, enquanto os estudos de adequação são úteis para mostrar se os resultados estão na direção esperada, o desenho quase-experimental apresenta a vantagem de verificar se os efeitos observados são próprios da intervenção, ampliando o potencial explicativo da avaliação em situações de pouco controle sobre o ambiente ^13^.

Em paralelo aos avanços metodológicos que ampliam a capacidade investigativa sobre as intervenções na rotina dos serviços, produções científicas sinalizam alternativas que possibilitam mudanças nos padrões de produção de bens e serviços de saúde no contexto em que se processam. A estratégia do apoio institucional e matricial propõe a horizontalização das relações entre trabalhadores e gestores, por meio do compartilhamento de saberes e práticas, fortalecendo a gestão e a operacionalização das ações no contexto organizacional ^14^.

Considerando a insuficiência de ações e os hiatos investigativos no contexto da produção dos dados para o fortalecimento da gestão e operacionalização do SIM, associados ao apoio institucional como alternativa para mudanças nas rotinas dos serviços de saúde, este estudo objetivou avaliar o SIM antes e depois da implementação de uma intervenção para aprimoramento do sistema, baseada em apoiadores institucionais.

## Métodos

Estudo avaliativo de uma intervenção para aprimoramento do SIM baseada no apoio institucional e matricial ^12^^,^^14^. Utilizou-se desenho quase-experimental a partir da análise quanto à influência do nível de implantação sobre os resultados observados no âmbito estadual e nas Regiões de Saúde, antes e depois da intervenção.

**Figura 1:**
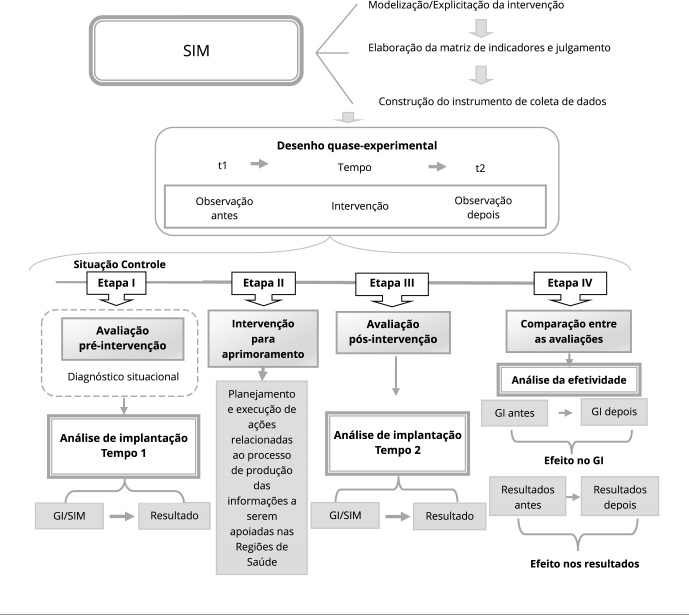
Síntese da avaliação da efetividade de intervenção para aprimoramento do Sistema de Informações sobre Mortalidade (SIM) em Pernambuco, Brasil.

Para o estudo pré-intervenção, procedeu-se a uma avaliação diagnóstica do SIM por meio da identificação dos principais problemas na produção de dados e do grau de sua implantação (GI) no contexto das Regiões de Saúde e dos respectivos municípios de abrangência, caracterizando a situação controle. A seguir, foi implementada a intervenção de aprimoramento do SIM, que consistiu em medidas direcionadas à correção das falhas operacionais do sistema, mediadas por apoiadores institucionais, valorizando-se as particularidades regionais e municipais. O estudo pós-intervenção repetiu os mesmos procedimentos adotados para a fase anterior ao seu desenvolvimento. Ao fim, compararam-se os dois momentos para averiguar as repercussões da intervenção de aprimoramento do SIM quanto à implantação e aos resultados alcançados. A Figura 1 mostra uma síntese das etapas de avaliação da efetividade para aprimoramento do SIM.

A intervenção e os dois momentos avaliativos ocorreram em Pernambuco, no Nordeste brasileiro, com área de 98.148,323km^2^. O estado está dividido em 12 Regiões de Saúde, que contêm 185 municípios ^15^.

GI: grau de implantação.

Para subsidiar a análise de implantação, foi atualizado o modelo lógico do SIM, previamente elaborado ^16^, mediante documentos regulamentadores do sistema, incluindo as *Portarias Ministeriais nº 116*, *nº 1.708* e *nº 47*, publicadas em 2009, 2013 e 2016, respectivamente; manuais de procedimentos e de preenchimento da Declaração de Óbito (DO), a exemplo do *Manual de Procedimentos do Sistema de Informações sobre Mortalidade*^17^ e *Manual de Instruções para o Preenchimento da Declaração de Óbito*^18^, amplamente divulgados pelo Ministério da Saúde como normas complementares à rotina de atividades no âmbito do SIM; e planos das ações desenvolvidas nos diferentes âmbitos de gestão do sistema para o alcance de metas estabelecidas, obtidos juntos à Secretaria Estadual de Saúde de Pernambuco (SES/PE) e desenvolvidos a partir de acordos interfederativos, como Programa de Qualificação das Ações de Vigilância em Saúde. O modelo lógico do SIM foi constituído de aspectos estruturais (recursos físicos e humanos) e de processos (atividades desenvolvidas), necessários para produção da informação no âmbito de cada componente do sistema: gestão; distribuição e controle; emissão e preenchimento; coleta; processamento; análise e divulgação das informações.

A partir do modelo lógico atualizado, elaborou-se a matriz de indicadores e julgamento. Para cada componente, estabeleceram-se indicadores de estrutura e processo, que expressaram o GI e de resultados. Os parâmetros de julgamento para o GI consideraram os documentos regulamentadores do SIM dos âmbitos federal ou estadual. Quando inexistentes, foram definidos empiricamente pelos pesquisadores, em consonância com a rotina do serviço, ou estipulados com base em normatizações, ainda que estas não explicitassem tais parâmetros ^7^.

A partir da matriz de indicadores foi elaborado um questionário, submetido para ajustes a um grupo de *experts*, técnicos e gestores estaduais do SIM. A versão final totalizou 65 questões, sendo 23 sobre aspectos estruturais e 42 sobre processuais, distribuindo-se entre os componentes do sistema.

Utilizaram-se dados primários, a partir da aplicação do questionário aos responsáveis pelo sistema nos municípios estudados, além de observação direta de aspectos estruturais e processuais em todas as secretarias de saúde entre novembro e dezembro de 2013, correspondendo ao estudo do período pré-intervenção, e um ano depois, nos mesmos meses de 2014, para a fase pós-intervenção. Também foram utilizados dados secundários, da base do SIM estadual, referentes aos óbitos ocorridos em 2012 e 2013, que se relacionaram, respectivamente, à fase anterior e posterior à intervenção, após o fechamento dos bancos de dados dos anos correspondentes. O Município de Itaíba foi o único excluído entre os 185 por não dispor de técnicos e gestores para operacionalizar e gerir as rotinas do sistema.

Os dados foram coletados por município e consolidados por componente e no âmbito integral do SIM, segundo unidade de análise estadual e Regiões de Saúde que constituíram os indicadores para a avaliação, considerando GI, resultados produzidos e influência do grau de implantação sobre os resultados.

Para verificar o GI do sistema, o cálculo de cada indicador considerou a razão entre os valores alcançados e esperados pelos municípios, referentes aos recursos físicos ou materiais e às atividades especificadas. A razão entre a soma dos valores alcançados e a soma dos valores esperados representou as dimensões de estrutura e processo. O GI de cada componente do SIM foi obtido mediante a razão entre o valor alcançado e esperado por essas dimensões, enquanto no âmbito integral do sistema calculou-se a razão entre a soma do GI alcançado e esperado por cada componente.

O GI foi classificado da seguinte forma: “implantado”, quando o valor alcançado em relação ao esperado correspondeu a percentuais que variaram de 80,0% a 100,0%; “parcialmente implantado”, de 60,0% a 79,9%; “incipiente”, de 40,0% a 59,9%; e “não implantado”, < 40,0%.

Os resultados produzidos pela intervenção com base na matriz de indicadores foram avaliados mediante a razão entre os valores alcançados e esperados para cada indicador e sua análise, a partir da observação do resultado por componente do sistema.

Para calcular a cobertura do SIM, indicador de resultado do componente coleta, o estudo se apoiou no posicionamento do Ministério da Saúde, que incorporou, a partir de 2008, a metodologia de Szwarcwald et al. ^10^ e Almeida & Szwarcwald ^11^ para estimar fatores de correção por estado e nível de adequação da informação nos municípios a partir de pesquisas de busca ativa de óbitos. Com esse suporte, a cobertura do SIM foi calculada por meio da razão entre o número de óbitos não fetais informados ao SIM e o número de óbitos estimados, multiplicada por 100.

A influência do GI sobre os resultados foi avaliada por meio das suas inter-relações, observando-se correspondência obtida em cada componente do SIM, com base no modelo lógico e na matriz de indicadores e julgamento do SIM.

A intervenção de aprimoramento do SIM, baseada no apoio institucional, direcionada às falhas identificadas na avaliação diagnóstica abordou o conjunto dos componentes descritos no modelo lógico. A intervenção contou com sete apoiadores institucionais para o nível estadual para mediar as ações nas regiões com deficiências e dois apoiadores por Região de Saúde, um com nível de escolaridade médio e outro com Ensino Superior, residentes onde as seguintes ações foram desenvolvidas: (1) encontros formativos: capacitações teórico-práticas sobre a normatização e operacionalização do SIM e análise de indicadores; (2) diagnóstico municipal: identificação dos problemas na produção da informação; (3) planejamento: elaboração dos planos de ação e correção de falhas identificadas; (4) execução das ações; e (5) monitoramento: acompanhamento das atividades e reajuste de ações inefetivas. Mais detalhes a respeito das etapas de avaliação, coleta das informações, indicadores, questões elaboradas e, em particular, sobre a intervenção para o aprimoramento do SIM estão contidos em Material Suplementar (https://cadernos.ensp.fiocruz.br/static//arquivo/supl-e00077523_8481.pdf).

Para avaliar as repercussões da intervenção de aprimoramento do SIM sobre a implantação e os resultados esperados, foram calculados o percentual de variação anual do GI e dos resultados alcançados no âmbito estadual e para cada Região de Saúde por componente e na perspectiva integral do sistema, antes e depois da intervenção.

Para favorecer a localização preferencial para as ações planejadas e identificar o comportamento dos dados do SIM antes e depois da intervenção, foram construídos mapas temáticos no software livre QGis, versão 2.18.9 (https://qgis.org/en/site/). Os mapas foram elaborados a partir dos indicadores de cobertura do SIM; proporção de DOs infantis com menos de 90% das variáveis do bloco IV preenchidas (razão entre o número de DOs infantis com menos de 90% das variáveis do bloco IV preenchidas e o total de óbitos infantis, multiplicada por 100) e proporção de óbitos por causa básica mal definida (razão entre o número de óbitos não fetais por causa básica mal definida e o total de óbitos não fetais, multiplicada por 100).

Este estudo foi aprovado pelo Comitê de Ética em Pesquisa em Seres Humanos do Instituto de Medicina Integral Prof. Fernando Figueira (Imip; CAAE 79846017.30000.5201).

## Resultados

### Análise comparativa do grau de implantação

O SIM foi classificado como “parcialmente implantado” no âmbito estadual nas avaliações pré (70,6%) e pós-intervenção (73,1%), com incremento de 3,6% entre os dois momentos, igualmente às Regiões de Saúde, ainda que houvesse reduções e incrementos superiores, com maior evidência na XII (21,1%), categorizando-se “implantada” (80,3%) depois das intervenções realizadas. Os componentes gestão, emissão e preenchimento e processamento foram ampliados de “parcialmente implantados” a “implantados” entre a avaliação pré e pós-intervenção: gestão nas III, VII e X nas regiões de saúde, com incremento médio de 11%; emissão e preenchimento, no âmbito estadual e nas II, IV, VI e XII; e processamento nas I, VIII e XII, variando positivamente de 26,8% a 28,3%, respectivamente (Tabela 1).

**Tabela 1:** Comparação entre o grau de implantação (GI) do Sistema de Informações sobre Mortalidade (SIM) no âmbito de gestão estadual e nas Regiões de Saúde por componentes e dimensão integral antes e depois da intervenção. Pernambuco, Brasil, 2012 e 2013.

Regiões de Saúde/Estudo	Gestão	Distribuição e controle	Emissão e preenchimento	Coleta	Processamento	Análise e divulgação	Síntese do GI
I							
Pré-intervenção	67,4	51,0	88,8	63,5	74,7	70,2	70,5
Pós-intervenção	74,4	62,0	96,1	65,0	80,0	67,7	76,2
Variação	10,3	21,6	8,2	2,4	7,1	-3,6	8,1
II							
Pré-intervenção	83,9	64,0	77,1	80,0	67,6	53,2	73,2
Pós-intervenção	86,1	58,0	81,3	77,1	65,3	46,9	72,3
Variação	2,6	-9,4	5,4	-3,6	-3,5	-11,8	-1,2
III							
Pré-intervenção	78,0	25,5	89,2	100,0	71,8	52,5	71,1
Pós-intervenção	85,1	42,7	68,1	93,8	70,1	59,2	72,3
Variação	9,1	67,6	-23,6	-6,3	-2,4	12,7	1,7
IV							
Pré-intervenção	67,9	67,5	72,2	83,3	75,3	61,5	70,5
Pós-intervenção	68,2	63,1	81,6	74,4	74,8	64,8	71,1
Variação	0,5	-6,5	13,1	-10,7	-0,7	5,4	0,8
V							
Pré-intervenção	76,7	51,0	78,3	75,0	68,3	50,9	69,3
Pós-intervenção	78,1	78,0	75,4	75,9	66,4	46,7	71,2
Variação	1,8	52,9	-3,7	1,2	-2,7	-8,3	2,7
VI							
Pré-intervenção	79,5	52,3	75,3	51,7	70,3	50,0	69,4
Pós-intervenção	73,1	47,7	94,0	97,0	67,9	51,4	70,6
Variação	-8,1	-8,8	24,8	87,6	-3,4	2,7	1,7
VII							
Pré-intervenção	76,2	17,1	78,6	61,9	68,7	68,4	67,0
Pós-intervenção	85,7	51,4	57,5	78,9	75,5	54,1	72,7
Variação	12,5	200,8	-26,8	27,5	9,9	-21,0	8,5
VIII							
Pré-intervenção	72,2	51,4	81,8	93,3	69,0	61,5	70,2
Pós-intervenção	76,2	62,9	90,7	84,2	84,8	73,7	79,2
Variação	5,5	22,3	10,9	-9,7	23,0	19,8	12,8
IX							
Pré-intervenção	80,3	23,6	80,6	100,0	81,3	66,2	74,8
Pós-intervenção	85,9	20,0	85,1	89,7	75,8	63,9	74,4
Variação	6,9	-15,3	5,6	-10,3	-6,8	-3,4	-0,5
X							
Pré-intervenção	74,3	60,0	75	91,2	75,3	50,0	71,9
Pós-intervenção	82,8	61,7	69,7	87,5	68,3	59,3	73,0
Variação	11,4	2,8	-7,1	-4,1	-9,3	18,6	1,6
XI							
Pré-intervenção	82,2	52,0	75,0	57,7	66,7	51,3	70,0
Pós-intervenção	84,4	40,0	65,0	92,9	72,5	53,7	71,8
Variação	2,7	-23,1	-13,3	60,9	8,7	4,7	2,6
XII							
Pré-intervenção	70,0	44,0	73,8	96,4	64,3	56,1	66,3
Pós-intervenção	77,8	78,0	93,5	100,0	82,5	59,6	80,3
Variação	11,1	77,3	26,8	3,7	28,3	6,3	21,1
Pernambuco							
Pré-intervenção	75,1	49,7	79,1	80,7	71,7	58,0	70,6
Pós-intervenção	78,6	57,0	80,6	83,0	72,7	58,5	73,1
Variação	4,7	14,6	1,9	2,8	1,4	0,8	3,6

A coleta “implantada” no estado e na maioria das Regiões de Saúde nas duas avaliações realizadas demonstrou expressiva variação positiva nas VI (87,6%) e XI (60,9%), que evoluíram de “incipientes” para “implantadas” na segunda avaliação. Distribuição e controle e análise e divulgação do SIM estadual permaneceram “incipientes” nos dois momentos, ainda que, entre as regiões, o primeiro apresentasse aumento no nível de implantação, e o segundo, discretas variações positivas (Tabela 1).

### Análise comparativa dos resultados

O componente gestão apresentou resultados em torno de 60% no âmbito estadual, com pouca variação entre os dois momentos. Diferentemente, as Regiões de Saúde mostraram resultados heterogêneos, com algumas reduções entre as avaliações, excetuando-se as IV, V, IX e XII. Entre as DOS distribuídas e cadastradas no SIM estadual, cerca de 90% das utilizadas foram informadas ao sistema, com resultados aproximados entre as regiões, especialmente depois da intervenção. Houve incremento na proporção de municípios que contabilizam os formulários distribuídos às unidades notificadoras, em especial nas Regiões de Saúde I (75%), VII e VIII (100%). A completude das DOS dos óbitos gerais permaneceu em torno de 50% no estado, com discreta variação positiva na maioria das Regiões de Saúde nos dois momentos. No entanto, a completude do bloco IV das DOs infantis ampliou de 66,8% para 78,9% no âmbito estadual, com incremento em todas as Regiões de Saúde, destacando-se as VII (96,2%) e VIII (65,8%). O SIM estadual revelou elevada proporção de óbitos com causa básica definida nas avaliações antes e depois da intervenção, com variações positivas na maioria das regiões, destacando-se a VIII, que ampliou 13,2% (Tabela 2).

**Tabela 2:** Comparação entre os resultados do Sistema de Informações sobre Mortalidade (SIM) no âmbito de gestão estadual e nas Regiões de Saúde por componentes e dimensão integral antes e depois da intervenção. Pernambuco, Brasil, 2012 e 2013.

Componentes/Indicadores de resultado/Estudo	Regiões de Saúde												Pernambuco
	I	II	III	IV	V	VI	VII	VIII	IX	X	XI	XII	
Gestão													
Percentual de municípios que monitoram ≥ 50% dos indicadores operacionais (cobertura, regularidade, causa básica de óbito definida) do SIM													
Pré-intervenção	90,0	45,0	45,5	100,0	15,0	100,0	85,7	85,7	72,7	100,0	100,0	10,0	69,6
Pós-intervenção	85,0	20,0	27,3	100,0	65,0	30,8	85,7	71,4	100,0	91,7	100,0	70,0	68,5
Variação	-5,6	-55,6	-40,0	0,0	333,3	-69,2	0,0	-16,7	37,5	-8,3	0,0	600,0	-1,6
Percentual de municípios com educação permanente realizada contabilizada													
Pré-intervenção	35,0	70,0	95,5	50,0	50,0	61,5	100,0	57,1	54,5	75,0	70,0	50,0	62,0
Pós-intervenção	30,0	55,0	95,5	81,3	70,0	53,8	42,9	42,9	45,5	75,0	60,0	40,0	62,5
Variação	-14,3	-21,4	0,0	62,5	40,0	-12,5	-57,1	-25,0	-16,7	0,0	-14,3	-20,0	0,9
Distribuição e controle													
Percentual de municípios com quantitativo de DO distribuída contabilizada													
Pré-intervenção	40,0	100,0	72,7	90,6	85,0	69,2	42,9	42,9	9,1	75,0	80,0	30,0	68,5
Pós-intervenção	70,0	100,0	59,1	90,6	85,0	76,9	85,7	85,7	9,1	58,3	70,0	20,0	71,7
Variação	75,0	0,0	-18,8	0,0	0,0	11,1	100,0	100,0	0,0	-22,2	-12,5	-33,3	4,8
Razão entre DO distribuída cadastrada no SIM e do DO utilizadas informadas no SIM													
Pré-intervenção	91,4	90,9	88,1	90,2	86,4	85,9	84,4	92,1	79,0	86,5	90,1	89,7	90,1
Pós-intervenção	92,0	93,2	84,6	90,8	88,3	86,9	83,1	90,2	81,7	90,2	91,3	89,9	90,8
Variação	0,6	2,5	-4,0	0,7	2,2	1,1	-1,6	-2,1	3,4	4,2	1,3	0,2	0,8
Emissão e preenchimento													
Percentual de DOs de óbitos infantis com ≥ 90,0% de preenchimento no bloco IV													
Pré-intervenção	75,7	62,7	53,5	74,0	59,3	62,0	44,4	53,3	60,2	50,0	55,8	68,2	66,8
Pós-intervenção	81,5	79,8	71,6	80,2	69,9	75,6	87,2	88,4	75,3	63,9	78,4	73,1	78,9
Variação	7,6	27,2	33,8	8,5	17,9	21,8	96,2	65,8	25,2	27,8	40,4	7,3	18,2
Percentual de DOs de óbitos gerais com ≥ 90,0% de preenchimento													
Pré-intervenção	59,6	45,8	48,5	54,4	40,9	41,8	50,1	43,3	23,9	30,8	32,5	52,2	51,7
Pós-intervenção	69,1	50,9	55,7	57,1	39,1	46,1	38,1	41,6	20,0	32,9	30,0	55,2	57,0
Variação	16,0	11,2	14,8	5,1	-4,5	10,2	-24,0	-4,1	-16,4	6,9	-7,6	5,7	10,2
Percentual de óbitos com causa básica definida													
Pré-intervenção	98,4	96,3	95,8	93,5	86,7	84,3	90,7	79,5	94,4	96,6	94,2	94,2	94,9
Pós-intervenção	98,4	96,2	96,3	93,2	90,4	85,7	93,6	90,0	95,1	95,7	91,8	97,7	95,6
Variação	0,0	-0,1	0,5	-0,2	4,4	1,7	3,1	13,2	0,7	-0,9	-2,5	3,7	0,7
Coleta													
Cobertura													
Pré-intervenção	98,3	103,0	97,9	99,7	105,6	105,9	96,4	92,9	104,7	99,8	97,7	101,9	100,0
Pós-intervenção	99,9	92,2	98,3	100,9	103,8	99,0	106,5	98,2	103,6	99,5	98,5	100,1	100,1
Variação	1,6	-10,4	0,5	1,2	-1,6	-6,5	10,5	5,7	-1,1	-0,3	0,8	-1,7	0,1
Percentual de municípios com ≥ 90,0% de cobertura o SIM													
Pré-intervenção	95,0	75,0	81,8	75,0	90,0	92,3	42,9	85,7	81,8	83,3	70,0	100,0	82,1
Pós-intervenção	85,0	60,0	81,8	87,5	90,0	84,6	71,4	71,4	81,8	83,3	70,0	80,0	80,4
Variação	-10,5	-20,0	0,0	16,7	0,0	-8,3	66,7	-16,7	0,0	0,0	0,0	-20,0	-2,0
Processamento													
Percentual de municípios com, no mínimo, um (1) envio de arquivo de transferência por mês no ano avaliado													
Pré-intervenção	97,1	93,3	91,7	95,3	95,4	91,0	97,6	94,0	93,9	87,5	98,3	97,5	94,3
Pós-intervenção	96,7	92,9	93,6	93,2	93,3	88,5	100,0	98,8	94,7	95,8	95,8	97,5	94,3
Variação	-0,4	-0,4	2,1	-2,2	-2,2	-2,8	2,4	5,1	0,8	9,5	-2,5	0,0	0,0
Percentual de DOs digitadas e enviadas no prazo oportuno, até 60 dias do mês de ocorrência													
Pré-intervenção	90,0	86,1	82,4	92,1	87,3	88,7	94,0	91,9	98,6	91,5	92,2	89,0	90,1
Pós-intervenção	92,4	81,8	81,7	94,5	88,6	84,5	102,5	96,1	97,2	94,5	93,2	90,7	91,3
Variação	2,6	-4,9	-0,8	2,6	1,4	-4,7	9,0	4,6	-1,4	3,3	1,1	1,9	1,4
Percentual de municípios com ≥ 80,0% das DOs digitadas e enviadas no prazo oportuno													
Pré-intervenção	85,0	50,0	54,5	75,0	80,0	69,2	71,4	85,7	90,9	83,3	90,0	80,0	73,9
Pós-intervenção	75,0	60,0	63,6	84,4	75,0	69,2	85,7	85,7	90,9	100,0	70,0	70,0	76,1
Variação	-11,8	20,0	16,7	12,5	-6,3	0,0	20,0	0,0	0,0	20,0	-22,2	-12,5	3,0
Percentual de óbitos de mulheres em idade fértil notificados ao SIM ≤ 30 dias da ocorrência													
Pré-intervenção	31,8	39,5	32,1	51,1	32,9	47,3	78,0	75,5	56,0	54,4	57,0	29,8	40,2
Pós-intervenção	47,8	45,0	39,6	54,4	55,9	47,6	87,5	79,0	52,5	50,0	72,0	27,4	50,9
Variação	50,5	13,9	23,6	6,6	69,8	0,7	12,2	4,7	-6,3	-8,1	26,3	-8,2	26,5
Percentual de óbitos de maternos notificados ao SIM ≤ 30 dias da ocorrência													
Pré-intervenção	18,8	*	22,2	0,0	0,0	*	100,0	100,0	62,5	*	0,0	0,0	26,2
Pós-intervenção	55,3	*	12,5	61,1	57,1	*	50,0	83,3	66,7	*	33,3	0,0	54,1
Variação	194,7	*	-43,8	NA	NA	*	-50,0	-16,7	6,7	*	NA	0,0	106,2
Percentual de óbitos fetais notificados ao SIM ≤ 30 dias da ocorrência													
Pré-intervenção	33,1	36,0	37,6	66,7	24,7	61,5	90,6	92,1	51,6	66,7	82,9	42,2	48,1
Pós-intervenção	55,7	59,7	63,4	76,1	65,9	70,7	77,3	90,8	57,1	58,3	90,0	54,5	64,5
Variação	68,5	65,9	68,6	14,2	167,2	14,9	-14,7	-1,4	10,8	-12,5	8,5	29,2	34,2
Percentual de óbitos infantis notificados ao SIM ≤ 30 dias da ocorrência													
Pré-intervenção	25,5	23,2	26,8	50,2	22,2	48,8	80,9	89,2	62,5	27,5	64,9	19,7	38,6
Pós-intervenção	49,5	50,6	50,7	68,4	50,6	57,0	74,4	95,9	62,3	58,3	70,3	31,3	57,4
Variação	94,5	117,9	89,6	36,4	127,9	16,8	-8,0	7,5	-0,3	112,1	8,2	59,1	48,8
Análise e divulgação													
Percentual de municípios com perfil epidemiológico elaborado													
Pré-intervenção	75,0	10,0	18,2	62,5	25,0	38,5	14,3	28,6	36,4	25,0	20,0	10,0	34,8
Pós-intervenção	50,0	15,0	13,6	65,6	35,0	38,5	28,6	28,6	18,2	33,3	10,0	40,0	34,2
Variação	-33,3	50,0	-25,0	5,0	40,0	0,0	100,0	0,0	-50,0	33,3	-50,0	300,0	-1,6
Percentual de municípios com boletim epidemiológico elaborado													
Pré-intervenção	50,0	10,0	13,6	50,0	15,0	38,5	14,3	57,1	36,4	33,3	30,0	30,0	31,5
Pós-intervenção	60,0	20,0	9,1	34,4	10,0	23,1	42,9	14,3	27,3	16,7	10,0	30,0	25,5
Variação	20,0	100,0	-33,3	-31,3	-33,3	-40,0	200,0	-75,0	-25,0	-50,0	-66,7	0,0	-19,0
Percentual de municípios que divulga os perfis epidemiológicos elaborados													
Pré-intervenção	55,0	10,0	18,2	59,4	25,0	30,8	14,3	28,6	36,4	25,0	20,0	10,0	31,5
Pós-intervenção	45,0	15,0	9,1	65,6	35,0	30,8	28,6	14,3	9,1	33,3	10,0	30,0	31,5
Variação	-18,2	50,0	-50,0	10,5	40,0	0,0	100,0	-50,0	-75,0	33,3	-50,0	200,0	0,0
Percentual de municípios que divulga os boletins epidemiológicos elaborados													
Pré-intervenção	45,0	10,0	13,6	46,9	15,0	30,8	14,3	57,1	36,4	33,3	30,0	30,0	29,9
Pós-intervenção	50,0	20,0	4,5	34,4	10,0	23,1	42,9	14,3	18,2	16,7	10,0	30,0	23,4
Variação	11,1	100,0	-66,7	-26,7	-33,3	-25,0	200,0	-75,0	-50,0	-50,0	-66,7	0,0	-21,8

DO: Declaração de Óbito; NA: não se aplica.

* Ausência de óbito materno no período de análise.

O componente coleta manteve cobertura acima de 90%, com discreta ampliação nas diferentes Regiões de Saúde entre os momentos estudados, ainda que se observasse redução na proporção de municípios com cobertura mínima de 90%. No componente processamento, a notificação oportuna dos óbitos de mulheres em idade fértil e dos óbitos fetais e infantis em até 30 dias da ocorrência apresentou incrementos superiores a 50%, 85% e 65%, respectivamente. Observaram-se na avaliação pós-intervenção os registros oportunos dos óbitos maternos em particular, nas IV, V e XI, ausentes na avaliação anterior. Os indicadores relacionados à análise e à divulgação apresentaram valores inferiores a 40%, com variações negativas no estado e nas Regiões de Saúde, com exceção das II, VII e XII, entre os dois momentos (Tabela 2).

### Análise comparativa da influência do GI sobre os resultados

O componente gestão apresentou correspondência entre o GI estadual e os indicadores de resultados nas duas avaliações realizadas. Entre as Regiões de Saúde, verificou-se ampliação da coerência entre o nível de implantação e a proporção de municípios que monitoram, no mínimo, 50% dos indicadores operacionais após a intervenção. O GI de emissão e preenchimento aumentou sua influência sobre a completude do bloco IV das DOs infantis e da proporção dos óbitos com causa básica definida em âmbito estadual nas diferentes Regiões de Saúde depois da intervenção. O nível de implantação da coleta manteve coerência com a cobertura geral de óbitos, expressa pela razão entre os eventos captados e estimados, nas duas avaliações, e evidenciou maior influência sobre a proporção de municípios com, pelo menos, 90% de cobertura nas Regiões de Saúde pós-intervenção.

O componente processamento do SIM estadual e das Regiões de Saúde revelou, respectivamente, manutenção e ampliação da coerência entre sua implantação e a proporção de municípios que enviam oportunamente os dados das DOs digitados, em comparação com as avaliações realizadas. O GI obtido pelo referido componente na avaliação pós-intervenção influenciou a notificação oportuna dos óbitos fetais no estado, em metade das Regiões de Saúde e na oportunidade da notificação dos óbitos infantis nas Regiões de Saúde IV, VII, VIII, IX e XI. Conforme a avaliação anterior à intervenção, não se observou correspondência entre o GI dos componentes distribuição e controle e análise e divulgação aos respectivos resultados alcançados em âmbito estadual na avaliação posterior, embora fossem verificadas coerências isoladas entre as distintas Regiões de Saúde.

### Localização preferencial para as ações planejadas e identificação do comportamento dos dados do SIM antes e após a intervenção

**Figura 2:**
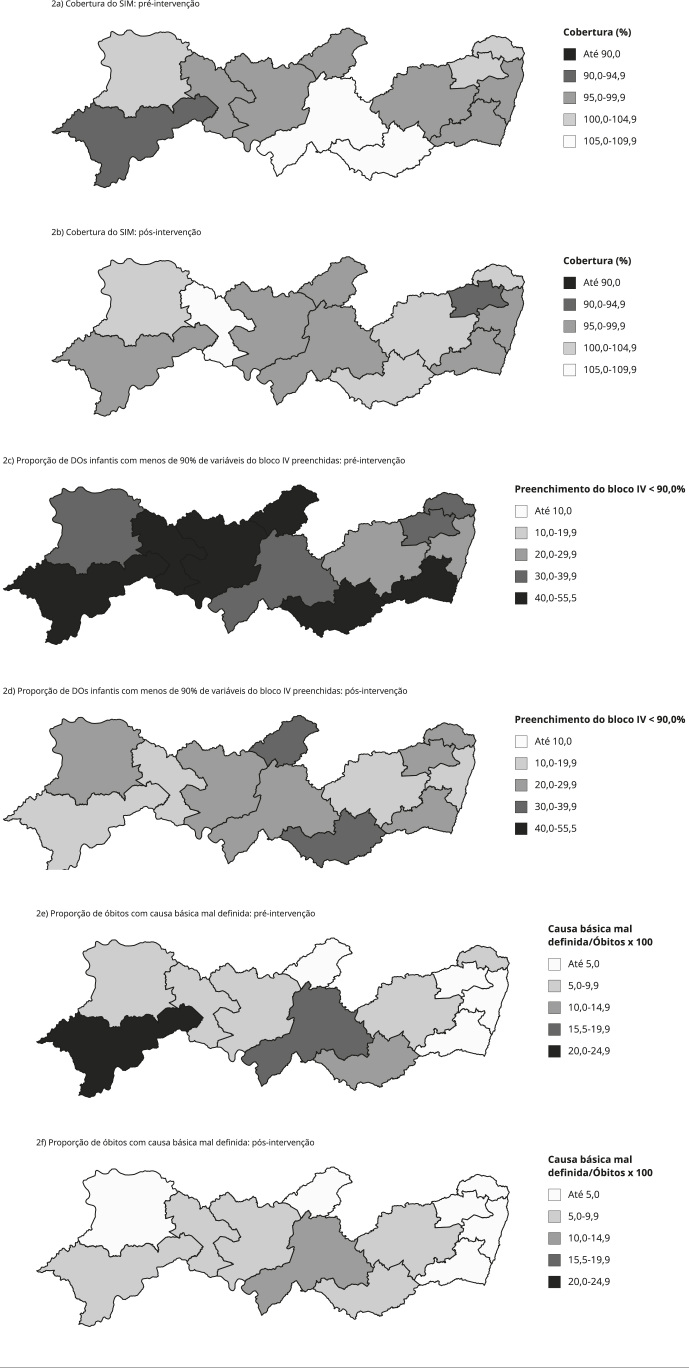
Mapas temáticos da cobertura do Sistema de Informações sobre Mortalidade (SIM), proporção de Declarações de Óbitos (DOs) infantis com menos de 90,0% de variáveis do bloco IV preenchidas e, proporção de óbitos com causa básica mal definida antes e depois da intervenção. Pernambuco, Brasil, 2012 e 2013.

Os mapas temáticos indicaram padrão mais homogêneo para os indicadores de cobertura e proporção de DOs infantis com menos de 90% das variáveis do bloco IV preenchidas entre as diferentes Regiões de Saúde. Quanto à proporção de óbitos com causa básica mal definida, duas Regiões de Saúde se destacaram com percentuais mais elevados em relação às demais. Depois da intervenção, observou-se mudança no padrão dos indicadores entre as Regiões de Saúde, com melhorias mais relevantes para a proporção de DOs infantis com menos de 90% das variáveis do bloco IV preenchidas. Entre as causas de óbito mal definidas, quatro Regiões de Saúde apresentaram melhora expressiva da cobertura, enquanto três exibiram piora, após a intervenção (Figura 2).

## Discussão

O estudo mostrou incremento de todos os componentes da cadeia de produção do SIM no ambiente de sua implantação ao se comparar os cenários antes e depois da intervenção de aprimoramento do sistema baseada em apoiadores institucionais. Igualmente, das 12 Regiões de Saúde, dez se beneficiaram com a intervenção, enquanto duas oscilaram negativamente, condicionadas, sobretudo, pelos componentes distribuição e controle; análise e divulgação; e coleta. Quase sempre, restrições no contexto da geração dos dados dos sistemas de informações vitais limitam o planejamento e a condução de ações capazes de influenciar seus resultados ^4^.

Intervenções focadas no processo de trabalho, considerando as diferenças entre Regiões de Saúde e municípios e valorizando suas especificidades, são capazes de interferir na produção da informação, possibilitando aumento da adesão às normas e repercussões na implantação do sistema ^7^^,^^11^^,^^19^. Esse enfoque na singularidade possibilitou classificar o SIM como implantado em âmbito integral na XII Região de Saúde e nos componentes emissão e preenchimento e coleta (nível estadual e mais da metade das Regiões de Saúde) e gestão e processamento (em algumas Regiões de Saúde).

Entre os resultados da intervenção, foram evidenciados, em geral, maior controle na distribuição das DOs às unidades notificadoras, melhoria da coleta e da notificação oportuna de óbitos maternos, fetais, infantis e de mulheres em idade fértil e da completude do bloco IV das DOs infantis, além da transmissão regular da informação às demais esferas de gestão. Estudos demonstram a ampliação da cobertura do SIM no estado, possibilitando o uso dos dados para o cálculo de indicadores de saúde com confiabilidade ^11^^,^^20^.

Quanto à cobertura do SIM, se destaca que diversas técnicas de estimação podem ser utilizadas, como: relacionamento entre registros; consistência de indicadores do próprio banco de dados; razão entre o número de eventos captados e estimados, mediante técnicas que utilizam dados do próprio sistema ou de outros; e busca ativa de eventos ^21^. O uso da razão entre o número de eventos captados e estimados a partir de fatores de correção produzidos com base nos dados de pesquisas de busca ativa de óbitos proposto pelo Ministério da Saúde se mostrou adequado. Ao cotejar os resultados sobre a cobertura do sistema para o Estado de Pernambuco referentes ao ano de 2012 ^11^ com os resultados deste estudo, observa-se melhoria desse indicador.

Para resolver os problemas que persistem na produção dos dados do SIM, aponta-se como imprescindível o fortalecimento da gestão dos formulários para sua melhor utilização e aperfeiçoamento do fluxo ^8^^,^^21^ e vigilância e assistência oportunas à saúde da mulher e da criança ^22^, associadas ao monitoramento e à avaliação para o aprimoramento dos dados. Dessa forma, a partir de ações transformadoras para a qualificação dos dados sobre eventos vitais, se viabiliza o *accountability* organizacional ^2^.

Vários países aceitaram os desafios expressos nos Objetivos de Desenvolvimento Sustentável (ODS) para 2030, que endossam a relevância de monitorar os indicadores de saúde, levantando questões para sua estimação na avaliação do alcance das metas ^23^^,^^24^. Um monitoramento adequado dos progressos alcançados por meio de intervenções salva-vidas garantiria a responsabilização dos governantes dos países em relação ao compromisso assumido com as Nações Unidas ^24^.

O reconhecimento da importância das estatísticas vitais favorece o desenvolvimento de ações para melhoria dos dados de registros vitais, priorizando áreas desfavorecidas com informações precárias ^10^^,^^25^. Entretanto, nem todos os países têm estatísticas de saúde completas e confiáveis ^26^. Assim, iniciativas que fortaleçam toda a cadeia de produção dos dados contribuem para o adequado acompanhamento dos indicadores e para o dimensionamento do esforço a ser empreendido para alcançar as metas.

Informações completas, precisas e oportunas contribuem para que gestores e trabalhadores da saúde ampliem sua capacidade de escolher e priorizar adequadamente intervenções para melhorar as políticas públicas, e a população participa por meio dos conselhos de saúde, favorecendo a tomada de melhores decisões, embora informações acuradas não assegurem a condução de boas decisões políticas ^27^. No Brasil, experiências locais exitosas de aperfeiçoamento dos sistemas de informação se multiplicam há décadas, particularmente em regiões que apresentam maiores limitações dos dados, com incorporação de algumas dessas iniciativas pelo Ministério da Saúde, por meio das secretarias de saúde dos estados e municípios ^8^^,^^10^^,^^28^.

Com relação ao SIM, a obtenção de melhores resultados entre os diferentes componentes do sistema reforça a potencialidade das intervenções processuais direcionadas aos problemas em cada etapa de produção da informação. Isso leva à ampliação das ações municipais, por vezes reduzidas à coleta e à transmissão de informações, e fortalece o papel da gestão local, com ressignificação das atribuições e do uso dos dados ^4^^,^^29^. Essa limitação do papel dos municípios tem relação com a concepção centralizadora e fragmentada dos sistemas de informação em saúde, historicamente constituídos para servir demandas nacionais, sinalizando dificuldades de descentralização ^30^. Em contraposição, foi nos municípios que emergiram intervenções úteis, viáveis e com larga chance de aplicabilidade, por considerarem os contextos sócio-históricos locais ^8^^,^^10^.

Ações relacionadas a gestão, emissão e preenchimento e coleta e processamento refletiram o fortalecimento local na adesão às rotinas operacionais do sistema, enquanto as relativas à análise e divulgação demonstraram maior dificuldade de integração das atividades analíticas, como anteriormente identificado ^30^^,^^31^. Ainda que o processo de descentralização tenha escolhido o município como espaço prioritário para execução e administração dos serviços de saúde, seu desenvolvimento deve estar relacionado à promoção de autonomia da gestão e do processo de trabalho, com perspectiva ao compartilhamento da capacidade decisória ^31^. Ao estimular capacidades técnica e administrativa e ampliar as rotinas do SIM, a intervenção fortaleceu a integralidade e a intensidade das ações nos âmbitos regional e municipal, favorecendo a operacionalização do sistema.

É provável que os avanços se relacionem à incorporação do apoio institucional na rotina dos serviços, estratégia que busca fomentar competências de gestão e de processo, potencializando o enfrentamento dos problemas experimentados na organização ^14^. Relatos sobre a incorporação desse dispositivo no âmbito da atenção primária à saúde possibilitaram a reestruturação de modos de fazer e pensar estagnados, por meio do acompanhamento qualificado das equipes ^32^. Sem estratégias adaptáveis aos contextos locais onde os dados são produzidos, associadas a uma boa comunicação que considere gestores e trabalhadores envolvidos, torna-se difícil a criação de ideias pertinentes para pessoas certas no momento certo e, consequentemente, as fontes de dados otimizadas que contribuiriam para a melhoria da saúde pública não seriam asseguradas ^27^.

O desenho quase-experimental utilizado viabilizou a investigação das intervenções no contexto de produção das informações e contribuiu para verificar, de maneira segura, se as repercussões da intervenção sobre a implantação e os resultados alcançados se deviam à intervenção e não a outros fatores próprios do ambiente ^13^. Por outro lado, a ocorrência da pandemia de COVID-19 e sua repercussão em todo o Sistema Único de Saúde (SUS) podem ter impactado negativamente a implantação dos sistemas de informação e, particularmente, o SIM ^33^. No Brasil, seus efeitos negativos se estenderam da diminuição nas coberturas vacinais à redução de notificações compulsórias, por possível refreamento nos diagnósticos ou perdas operacionais causadas pela pandemia aos programas de vigilância ^33^.

Entre os limites, existiu possibilidade de familiarização com o questionário entre os participantes da pesquisa, com facilitação das respostas no segundo momento; interação entre intervenção e fatores contextuais, afetando seus resultados; além do viés associado à aferição dos efeitos, mediante aumento do domínio dos observadores sobre o instrumento de medida entre o início e o fim do estudo ^13^. O limite orçamentário condicionou o número de apoiadores institucionais nas Regiões de Saúde, com diferentes tamanhos e portes populacionais, interferindo no tempo dedicado ao desenvolvimento da intervenção.

Para minimizar as limitações, buscou-se ampliar a validade interna do estudo por meio da elaboração e explicitação do modelo lógico e da matriz de julgamento para observar o grau de conformidade da intervenção, identificando as fragilidades e potencialidades ^12^.

O estudo ampliou as possibilidades investigativas do SIM em relação às avaliações comumente realizadas ao analisar toda a cadeia de produção da informação no contexto organizacional, permitindo identificar os problemas na geração dos dados e subsidiar o planejamento de ações direcionadas às falhas identificadas. A incorporação da estratégia do apoio institucional repercutiu nos resultados, apresentando-se como alternativa no enfrentamento das dificuldades experimentadas na rotina dos serviços, ao implicar mudanças no processo de trabalho e na capacidade de gestão.
